# Correlation between ocular parameters and amplitude of accommodation

**DOI:** 10.4103/0301-4738.71680

**Published:** 2010

**Authors:** Lekha Mary Abraham, Thomas Kuriakose, Viswanathan Sivanandam, Nithya Venkatesan, Ravi Thomas, Jayaprakash Muliyil

**Affiliations:** Department of Ophthalmology, Christian Medical College, Vellore, India; 1 Queensland Eye Institute, South Brisbane, Queensland, Australia; 2 Department of Community Health, Christian Medical College, Vellore, India

**Keywords:** Amplitude of accommodation, anterior chamber depth, axial length, lens thickness, presbyopia

## Abstract

**Aim:**

To study the relationship between ocular parameters and amplitude of accommodation (AA) in the peri-presbyopic age group (35–50 years).

**Materials and Methods:**

Three hundred and sixteen right eyes of consecutive patients in the age group 35–50 years, who attended our outpatient clinic, were studied. Emmetropes, hypermetropes and myopes with best-corrected visual acuity of 20/20, J1 in both eyes were included. The AA was calculated by measuring the near point of accommodation. The axial length (AL), central anterior chamber depth (CACD) and lens thickness (LT) were also measured.

**Results:**

There was moderate correlation (Pearson’s correlation coefficient *r* = 0.56) between AL and AA as well as between CACD and AA (*r* = 0.53) in myopes in the age group 35–39 years. In the other age groups and the groups taken as a whole, there was no correlation. In hypermetropes and emmetropes, there was no correlation between AA and the above ocular parameters. No significant correlation existed between LT and AA across different age groups and refractive errors.

**Conclusion:**

There was no significant correlation between AA and ocular parameters like anterior chamber depth, AL and LT.

The unique ability of the eye to vary the refractive power of the lens and to focus on things at a range of distances is called accommodation. The alteration in the curvature of the lens along with anterior movement of lens-iris diaphragm is currently considered to be the key factor responsible for the change in refraction.[1,2] The reduction of this ability in which the near point recedes further away from comfortable reading distance is called presbyopia. There is continuing research to understand this process and correct this affliction that affects each and every person at the peak of their productive life.[3] With an aging population, the proportion of people above 40 years is on the rise. This will therefore have public health and economic implications.

Though presbyopia is a universal phenomenon, the age at which people become symptomatic varies. It is well known that myopes seek help for presbyopic symptoms much later than emmetropes and hypermetropes. However, the exact reason for this phenomenon is still not very clear. Though the removal of distance correction while doing near work[4] and the under correction prescribed for myopes[5] could contribute to this delay, there are studies showing that myopes have higher amplitudes of accommodation (AAs) which they preserve for longer periods.[6,7]

We are also aware that myopes generally have deeper anterior chambers and longer axial lengths (ALs).[7,8] However, it is not known whether these ocular features facilitate the movement of lens-iris diaphragm through larger distances. There are a few data on the ocular parameters and their relation to presbyopia. The aim of this study was to determine whether central anterior chamber depth (CACD), AL and lens thickness (LT) affect the AA.

## Materials and Methods

In this cross-sectional study, all patients who attended our outpatient department between July 1999 and July 2000 were considered, provided they fulfilled the following inclusion and exclusion criteria.

Inclusion criteria:

1. Patients between 35 and 50 yrs of age.
2. Best corrected visual acuity of 20/20, J1 in both eyes.

Exclusion criteria:

1. Any ocular pathology including cataract ≥ grade NO1, NC1, C1, P1 according to LOCS III cataract classification system.[9]
2. Spherical corrections of more than 6.00 diopter sphere (Dsph) of hypermetropia or myopia.
3. Cylindrical correction of more than 0.75 diopter cylinder (Dcyl).
4. History of diabetes mellitus, ocular trauma, childhood diphtheria, glaucoma, retinal photocoagulation and uveitis.
5. History of prolonged use of topical cycloplegics, phenothiazines, tricyclic antidepressants and antivertigo drugs.

An emmetropic eye is defined as one in which the spherical correction is less than or equal to ±0.25 diopters (D) after undilated retinoscopy and subjective refraction. Those with spherical correction of more than or equal to +0.50 D were considered hypermetropes and those with a spherical correction of more than or equal to −0.50 D were considered myopes. The completed age in years was taken for age calculation. The best-corrected visual acuity was obtained after undilated retinoscopy, subjective refraction and a duochrome test.

After a written informed consent, a complete ophthalmic examination was performed on all patients before 11:00 a.m. With full distance correction in the trial frame placed 15 mm from the eye, near point of accommodation (NPA) was measured, one eye at a time, using the RAF (Royal Air Force) rule. The NPA was measured with the patient trying to read the smallest letter (N5) on the RAF rule target. With the RAF rule in place, the target was moved from 50 cm to the point where the last line became slightly blurred. Then, the target was slowly pushed back till the last line could just be clearly read. This point was taken as the NPA. No correction for inter-pupillary distance was attempted because the correction factor was too small to make any significant difference in the diopteric value of amplitude in this age group. If the NPA was more than 50 cm, a +1.00 D spherical lens was added to the trial frame and the near point measured again. One diopter was subtracted from the reciprocal of the measured NPA to get the actual amplitude in these patients. Reciprocal of the NPA in meters is the AA.

AL, CACD and LT were measured using A scan (8–10 MHz Tomey AL 1000). CACD was measured by keeping the A scan probe gently on the cornea with the center of the pupil as the reference point and the patient fixing at a distant nonaccommodative target. For each of the above parameters, two values with a difference of 0.2 mm or less were taken and averaged.

Two of the authors (V.S. and N.V.) took all the measurements. The kappa value for inter-observer agreement for these two examiners was 0.82. We used the Pearson’s correlation coefficient (*r*)[10] to determine the correlation between AA and ocular parameters in all the refractive groups. Analysis of covariance (ANCOVA) was also performed using SPSS 9.0 on the data, to arrive at the correlation while controlling for age. Taking the correlation between CACD and AA as the primary outcome, the power of the study was also calculated.

## Results

Three hundred and sixteen subjects were included in the study. Of these 144 were males and 172 were females. Right eyes were used for analysis. One hundred and sixty-two eyes were emmetropic, 104 eyes were hypermetropic and 50 were myopic. The distribution of the number of eyes studied, their age groups and refractive errors are given in Table 1.

**Table 1:** Distribution of patients across age groups and oprefractive errors

Age (years)	E	H	M	Total
35–39	43	16	16	75
40–44	73	43	16	132
45–50	46	45	18	109
Total	162	104	50	316

E: Emmetropes, H: Hypermetropes, M: Myopes

Mean CACD across all the age groups was found to be 3.18 mm in emmetropes, 2.86 mm in hypermetropes and 3.36 mm in myopes, as given in Table 2. The normal CACD is 3.15 mm (2.6–4.4).[11] With Student’s t test, statistically significant differences between emmetropes and hypermeropes (*P* = 0.045), hypermetropes and myopes (*P* = 0.015) and emmetropes and myopes (*P* = 0.034) were observed. Mean ALs [Table 3] across all the age groups were 22.86, 22.44 and 23.26 mm in emmetropes, hypermetropes and myopes, respectively. There was a statistically significant difference between the AL in hypermetropes and emmetropes (*P* = 0.038) and also between hypermetropes and myopes (*P* = 0.012). There was no statistically significant difference in the AL between emmetropes and myopes (*P* = 0.09).

**Table 2:** Mean central anterior chamber depth in millimeters

Age (years)	Emmetropes	Hypermetropes	Myopes
35–39	3.20 (CI: 3.19–3.21)	2.91 (CI: 2.77–3.05)	3.30 (CI: 3.15–3.44)
40–44	3.22 (CI: 3.18–3.26)	2.76 (CI: 2.68–2.84)	3.43 (CI: 3.32–3.51)
45–50	3.04 (CI: 2.98–3.10)	2.91 (CI: 2.86–2.96)	3.33 (CI: 3.24–3.42)

CI: Confidence interval

**Table 3:** Mean axial length in millimeters

Age (years)	Emmetropes	Hypermetropes	Myopes
35–39	22.78 (CI: 22.67–22.89)	22.34 (CI: 22.12–22.56)	23.44 (CI: 23.00–23.88)
40–44	22.95 (CI: 22.83–23.07)	22.30 (CI: 22.14–22.46)	22.94 (CI: 22.72–23.12)
45–50	22.83 (CI: 22.70–22.96)	22.62 (CI: 22.50–22.74)	23.42 (CI: 23.01–23.83)

The LT [Table 4] increases with increasing age. Myopes seem to have thinner lenses but this was not statistically significant. AA across all the age groups and refractive errors are given in Table 5. There was moderate correlation (Pearson’s correlation coefficient *r* = 0.56) between AL and AA as well as between CACD and AA (*r* = 0.53) in myopes in the age group of 35–39 years. In the other age groups or refractive groups there was no correlation. There was no significant correlation between LT and AA.

**Table 4:** Mean LT in millimeters

Age (years)	Emmetropes	Hypermetropes	Myopes
35–39	4.36 (CI: 4.25–4.47)	4.39 (CI: 4.24–4.54)	4.34 (CI: 4.26–4.42)
40–44	4.41 (CI: 4.32–4.50)	4.47 (CI: 4.35–4.59)	4.35 (CI: 4.28–4.42)
45–50	4.58 (CI: 4.46–4.70)	4.45 (CI: 4.34–4.56)	4.40 (CI: 4.24–4.56)

**Table 5:** Mean AA in diopters

Age (years)	Emmetropes	Hypermetropes	Myopes
35–39	2.75 (CI: 2.65–2.85)	2.73 (CI: 2.52–2.94)	3.71 (CI: 3.21–4.41)
40–44	2.33 (CI: 2.24–2.42)	1.84 (CI: 1.72–1.96)	2.63 (CI: 2.45–2.81)
45–49	1.54 (CI: 1.47–1.61	1.62 (CI: 1.53–1.71)	1.72 (CI: 1.60–1.84)

ANCOVA also did not show any significant correlation between AA and ocular parameters in the three refractive groups. The significance levels (*P*) for the correlation between AA and CACD were 0.616, 0.425 and 0.988 for emmetropes, hypermetropes and myopes, respectively. The *P* values for correlation of AA versus AL were 0.981, 0.582 and 0.276 and those of AA versus LT were 0.845, 0.107 and 0.785, for emmetropes, hypermetropes and myopes, respectively.

The power of the study was calculated on the moderate correlation between CACD and AA using the formula; *n* = {(zα+ zβ)^2^/[*r*^2^/(1 – r^2^)]}+2, where *n* = 16 (myopes in the age group of 35–39 years; Table 1) and *r* = 0.53. The β error was 0.37 with a power of 65%.

## Discussion

Among the three refractive groups, myopes develop presbyopic symptoms much later than hypermetropes and emmetropes. Myopes are also expected to have deeper anterior chambers compared to the other refractive groups. This study which focused on whether anatomical parameters have a role in preserving accommodation was a part of another study dealing with AA in the peri-presbyopic age group, viz., 35–50 years, which showed that myopes preserve their AA for longer periods.[6]

The lower mean AA in the population studied compared to the younger age groups and the low variability between the refractive groups was precisely the property that we wanted to take advantage of by studying this population. After the age of 50 years, lenticular changes become more universal especially in tropical areas, thus reducing the variability to a level where it becomes difficult to make out the differences between the groups.

The study was primarily carried out to see if anterior chamber depth variation was one of the reasons for the varying preservation of accommodation. Though myopes have deeper anterior chambers compared to other refractive groups, lack of correlation of CACD with AA suggests that this does not contribute significantly to the increased AA in myopes.

The LT increases with increasing age. The reason for calculating LT and its relationship to AA was to see if baseline LT affected the ability of the lens to change its shape during the process of accommodation. Our data suggest that no such relationship existed. Even a significant difference in AL did not affect the AA.

Even though the study population had variations of up to 3 D of accommodation within the 35–50 year age interval, we could not find any correlation between AA and anterior chamber depth in the study group as a whole. Theoretically, the anterior movement of the lens can increase the vergence of light. In a small study on 20 subjects (10 emmetropes and 10 myopes), Bolz *et al*. have found a linear correlation between refractive and axial biometric changes of the anterior segment during accommodation.[12] However, our results suggest that the accommodative process is not related to anterior chamber depth in the peri-presbyopic age group.

We wanted to take advantage of the lower AA in the group we studied, but it became a limitation when we tried to calculate the correlation between AA and ocular parameters. The limited range of AA may be one of the reasons why we did not find any correlation between the two. Since the power of the study was only about 65%, a larger sample size may have revealed a correlation. Using contact methods to study ocular parameters has its disadvantages and thus non contact methods like the Scheimpflug camera would have been better.

Future studies should use larger number of patients, especially myopes, if the same age group of patients is studied to find similar correlations. Younger age group subjects with larger AA are a better option. Presbyopia is still not a fully solved puzzle and continuous research to fully understand the physiology is critical in developing the strategies to prevent or treat this condition.
